# Gfi1 upregulates c-Myc expression and promotes c-Myc-driven cell proliferation

**DOI:** 10.1038/s41598-020-74278-4

**Published:** 2020-10-13

**Authors:** Yangyang Zhang, Fan Dong

**Affiliations:** Department of Biological Sciences, University of Toledo, Toledo, OH 43606 USA

**Keywords:** Cell growth, Ubiquitylation, Molecular medicine, Haematological cancer

## Abstract

Gfi1 is a zinc-finger transcriptional repressor that plays an important role in hematopoiesis. When aberrantly activated, Gfi1 may function as a weak oncoprotein in the lymphoid system, but collaborates strongly with c-Myc in lymphomagenesis. The mechanism by which Gfi1 collaborates with c-Myc in lymphomagenesis is incompletely understood. We show here that Gfi1 augmented the expression of c-Myc protein in cells transfected with c-Myc expression constructs. The N-terminal SNAG domain and C-terminal ZF domains of Gfi1, but not its transcriptional repression and DNA binding activities, were required for c-Myc upregulation. We further show that Gfi1 overexpression led to reduced polyubiquitination and increased stability of c-Myc protein. Interestingly, the levels of endogenous c-Myc mRNA and protein were augmented upon Gfi1 overexpression, but reduced following Gfi1 knockdown or knockout, which was associated with a decline in the expression of c-Myc-activated target genes. Consistent with its role in the regulation of c-Myc expression, Gfi1 promoted Myc-driven cell cycle progression and proliferation. Together, these data reveal a novel mechanism by which Gfi1 augments the biological function of c-Myc and may have implications for understanding the functional collaboration between Gfi1 and c-Myc in lymphomagenesis.

## Introduction

The zinc-finger (ZF) transcriptional repressor Gfi1 is critically involved in the regulation of hematopoiesis^1,2^. Targeted deletion of *Gfi1* severely impairs T cell development and reduces B cell numbers in mice. Gfi1 also supports neutrophil development and suppresses the alternative development towards monocytes/macrophages. In addition, Gfi1 has been shown to regulate the self-renewal and survival of hematopoietic stem cell (HSC)^3–5^. *Gfi1*^*-/-*^ HSCs are hypersensitive to stress-induced apoptosis and their ability to reconstitute long-term hematopoiesis in recipient mice is defective.

When overexpressed, Gfi1 has been shown to act as a weak oncoprotein in the lymphoid system. It has been shown that Gfi1 overexpression in B and T lymphocytes abrogated cell cycle block following growth factor withdrawal^6–8^. Increased expression of Gfi1 in T cells was associated with T cell lymphoma in transgenic mice^9–11^. In contrast, mice were cured from acute lymphoblastic leukemia (ALL) following *Gfi1* ablation^12^. It has also been shown that Gfi1 functionally collaborated with c-Myc in lymphomagenesis. Combinational overexpression of Gfi1 and c-Myc considerably accelerated lymphoma development whereas loss of Gfi1 led to lymphoma regression in *Eμ-Myc* transgenic mice^9–12^, indicating an important role of Gfi1 in the initiation and maintenance of c-Myc-driven lymphoma development. Gfi1 expression is increased in human lymphoma and acute lymphoblastic leukemia cells^12–14^.

The mechanisms by which Gfi1 collaborates with c-Myc in lymphomagenesis is incompletely understood. We previously showed that Gfi1 and c-Myc form a ternary complex with Miz-1, and act in collaboration to repress CDK inhibitors *p15*^*INK4B*^, *p21*^*Cip1*^ and *p27*^*Kip1*^^15,16^, providing a potential explanation for Gfi1 involvement in Myc-induced lymphomagenesis. The expression of c-Myc is strictly regulated in normal lymphoid cells, but c-Myc is frequently overexpressed in lymphoma cells, usually resulting from gene amplifications and chromosomal translocations^17,18^. For instance, *MYC* translocations leading to its overexpression are a defining feature of Burkitt lymphoma. c-Myc overexpression may also occur as a result of stabilization of the highly unstable c-Myc protein, which is rapidly degraded via the ubiquitin–proteasome pathway^19,20^. In this paper, we show that Gfi1 increased c-Myc protein stability and mRNA level, leading to augmented c-Myc expression and cell proliferation driven by c-Myc. Our data may reveal a novel mechanism of the functional collaboration between Gfi1 and c-Myc in lymphomagenesis.

## Results

### Gfi1 upregulates c-Myc protein level

**Figure 1:**
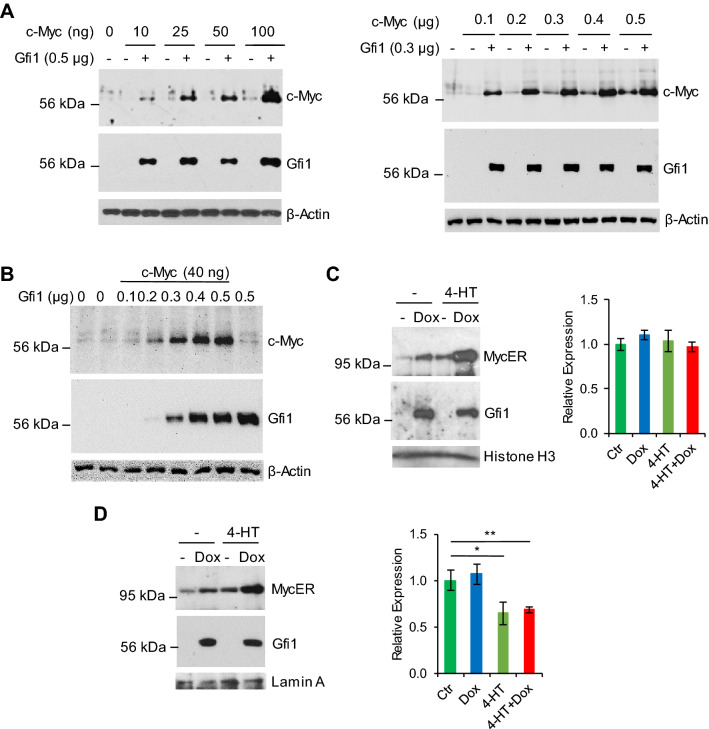
Gfi1 upregulates the expression of c-Myc protein. Hela cells were transfected with fixed amounts of Gfi1 and increasing amounts of c-Myc (**A**), or with fixed amount of c-Myc and increasing amounts of Gfi1 (**B**). BaF/MycER/Gfi1 (**C**) and 32D/MycER/Gfi1 (**D**) cells were treated with Dox, 4-HT or both for 24 h. The expression of indicated proteins was examined by Western blot analysis (left panels). Full-length blots are presented in Supplementary Fig. 6. MycER mRNA levels were quantitated by qRT-PCR (right panels). Data are shown at mean ± SD.

The expression of c-Myc is tightly regulated in normal lymphoid cells. Notably, most lymphomas with MYC overexpression originate from cells that do not normally express MYC, suggesting that these tumors have developed additional oncogenic events to disrupt the MYC regulatory mechanisms^18^. In our previous studies^15,16^, we noticed that Gfi1 significantly increased the protein level of c-Myc transiently expressed in Hela cells. As Gfi1 collaborates with c-Myc in lymphomagenesis, we further examined the role of Gfi1 in regulating c-Myc expression. Hela cells were transiently transfected with increasing amounts of c-Myc without or with fixed amount of Gfi1 or vice versa. The expression of c-Myc protein was examined by Western blot analysis. As shown in Fig. 1A,B, Gfi1 markedly augmented the level of c-Myc protein in all the experimental conditions, indicating that Gfi1 was required for efficient expression of c-Myc protein in Hela cells. The effect of Gfi1 on c-Myc expression was specific as Gfi1 did not upregulate the expression of other transcription factors, including AML1, C/EBPε, STAT5 and Miz-1 (supplementary Fig. 1S).

We next addressed whether Gfi1 augmented c-Myc expression in hematopoietic cells. We generated Ba/F3 (BaF/MycER/Gfi1) and 32D (32D/MycER/Gfi1) cells that stably expressed c-MYC/estrogen receptor ligand binding domain fusion protein (MycER) and inducibly expressed Gfi1 in response to doxycycline (Dox). The Dox-induced expression of Gfi1 in Ba/F3 cells was lower than the expression of endogenous Gfi1 in human myeloid leukemic HL60 and U937 cells, which expressed relatively high levels of Gfi1 (supplementary Fig. 2S). BaF/MycER/Gfi1 and 32D/MycER/Gfi1 cells were treated with 4-hydroxytamoxifen (4-HT), Dox or both for 24 h prior to evaluation of protein expression by Western blot analysis. As expected, treatment with 4-HT increased the MycER protein levels in the two cell lines (Fig. 1C, D). Irrespective of 4-HT treatment, induction of Gfi1 expression with Dox resulted in a significant increase in MycER protein level in the nuclei of the cells. Dox and 4-HT had no significant effect on the expression of MycER mRNA in Ba/F3 although 4-HT appeared to inhibit MycER mRNA expression in 32D cells. Together, these results demonstrated that Gfi1 upregulated MycER protein expression in hematopoietic cells.

**Figure 2:**
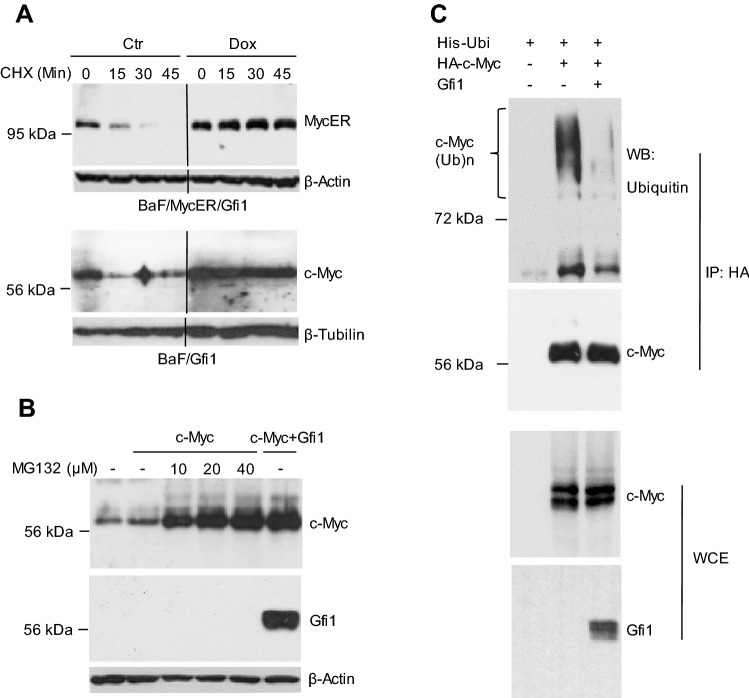
Gfi1 reduces the polyubiquitination and increases the stability of c-Myc protein. (**A**) BaF/MycER/Gfi1 (upper panels) and BaF/Gfi1 (lower panels) cells were untreated (Ctr) or treated with Dox for 24 h, followed by treatment with cycloheximide (CHX; 30 μg/ml) for different times. The blots were cropped as indicated by the vertical lines. (**B**) Hela cells were transfected as indicated, and then treated with MG132 at different concentrations for 6 h prior to preparation of whole cell extracts. (**C**) Hela cells were transfected as indicated and treated with MG132 for 6 h. Whole cell extracts (WCE) were subjected to immunoprecipitation using the HA antibody under the denaturing condition. c-Myc expression and polyubiquitination were examined by Western blot analysis. Full-length blots are presented in Supplementary Fig. 7.

### Gfi1 reduces the polyubiquitination of, and increases the stability of c-Myc protein

c-Myc protein is highly unstable with a half-life of less than 30 min in non-transformed cells and is rapidly degraded mainly through the ubiquitin–proteasome pathway^19,20^. To examine whether Gfi1 stabilized c-Myc protein, BaF/MycER/Gfi1 cells were treated with Dox for 24 h to induce Gfi1 expression, followed by treatment with cycloheximide (CHX) to block protein synthesis. MycER protein level rapidly declined and became barely detectable at 30 min in BaF/MycER/Gfi1 cells untreated with Dox, but was stable for at least 45 min in Dox-treated cells (Fig. 2A). To examine whether Gfi1 stabilized endogenous c-Myc, we generated Ba/F3 cells expressing the Dox-inducible Gfi1 (BaF/Gfi1). As shown in Fig. 2A, Dox-induced expression of Gfi1 (see Fig. 5A) led to the significantly increased stability of endogenous c-Myc protein. Interestingly, when Hela cells transfected with c-Myc alone were treated with different amounts of proteasome inhibitor MG132, the expression of c-Myc protein increased to a level similar to that in cells transfected with both c-Myc and Gfi1 (Fig. 2B), suggesting that Gfi1 may inhibit c-Myc degradation mediated by the ubiquitin–proteasome pathway. We further performed ubiquitination assay to assess the effect of Gfi1 on c-Myc polyubiquitination. As shown in Fig. 2C, co-expression of c-Myc and Gfi1 markedly reduced c-Myc polyubiquitination in Hela cells. Together, these data strongly suggest that Gfi1 stabilized c-Myc protein by reducing its polyubiquitination.

### The N-terminal SNAG domain and C-terminal ZF domains of Gfi1 are required for c-Myc upregulation

**Figure 3:**
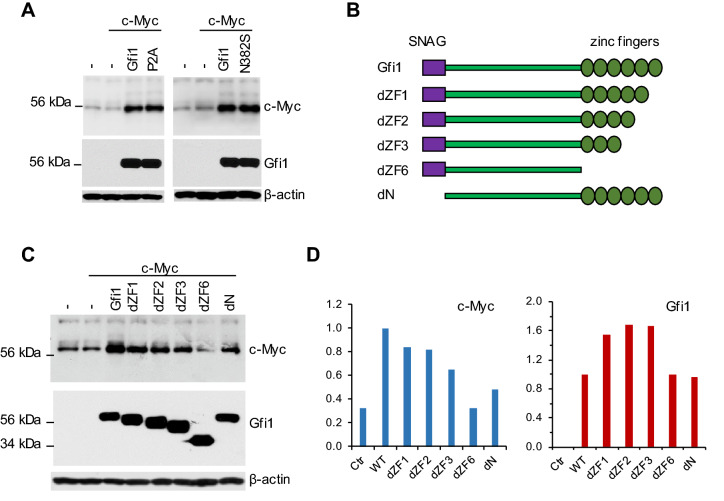
The SNAG and C-terminal ZF domains, but not the DNA binding and transcriptional repression activities of Gfi1, are required for c-Myc upregulation. (**A**) Hela cells were transfected with c-Myc alone or together with Gfi1, the P2A (left panels) or N382S mutant (right panels). (**B**) Schematic diagrams of Gfi1 and the different truncation mutants. (**C**) Hela cells were transfected with c-Myc along with the different forms of Gfi1. The expression of the indicated proteins was examined by Western blot analysis. Full-length blots are presented in Supplementary Fig. 8. (**D**) The results shown in C were quantitated using ImageJ and normalized based on the expression levels of β-actin.

We examined which activities or domains of Gfi1 were required for c-Myc upregulation. The Gfi1 N382S mutant is defective in DNA binding whereas the P2A mutant lacks the repressor activity^21–23^. As shown in Fig. 3A, both Gfi1 mutants upregulated c-Myc expression as efficiently as the wild type (WT) Gfi1 in Hela cells. Gfi1 has an N-terminal SNAG domain and 6 ZF domains at its C-terminus that are involved DNA binding and protein–protein interactions. We generated the Gfi1 mutants lacking the N-terminal SNAG domain or with progressive truncation of the C-terminal ZF domains (Fig. 3B). Deletion of the SNAG domain markedly diminished c-Myc upregulation by Gfi1. Interestingly, progressive truncation of the C-terminal ZF domains resulted in the gradual loss of the ability of Gfi1 to upregulate c-Myc, which was associated with an increase in the expression of the mutant Gfi1 proteins (Fig. 3C, D). Thus, both the SNAG domain and the ZF domains, but not the DNA binding and repressor activities of Gfi1, were required for c-Myc upregulation.

### Fbxw7 and Skp2 are not the major E3 ubiquitin ligases involved in c-Myc upregulation by Gfi1

It has been shown that Gfi1 is also polyubiquitinated and degraded through the ubiquitin–proteasome pathway, which takes place at its C-terminal ZF domains^24^, consistent with the augmented expression of the C-terminally truncated Gfi1 mutants. We addressed the possibility that Gfi1 might upregulate c-Myc protein expression by competing with c-Myc for a common E3 ubiquitin ligase(s). A number of c-Myc E3 ubiquitin ligases, including Fbxw7, Skp2, CHIP,FBX29, FBXO32, PirH2, TRIM32 and Truss have been reported to reduce c-Myc protein expression^19,20^. Interestingly, Gfi1 has been identified as a substrate of Fbxw7^25^. c-Myc degradation catalyzed by Fbxw7 is dependent on c-Myc threonine 58^26^. We therefore examined the effect of Gfi1 on the expression of c-Myc T58A, which is resistant to Fbxw7-mediated degradation. As shown in supplementary Fig. S3A, the expression of c-Myc T58A was upregulated by Gfi1 in Hela cells. Furthermore, Gfi1 upregulated the level of c-Myc transiently expressed in *FBXW7*^*-/-*^ HCT116 cells (supplementary Fig. S3B). Together, these data suggested that Fbxw7 may not play a major role in Gfi1-mediated stabilization of c-Myc protein.

Skp2 is another major regulator of c-Myc protein stability. Skp2 binds to the MBII and basic helix-loop-helix leucine zipper (bHLH-LZ) domains of c-Myc^27,28^. We examined whether Gfi1 upregulated the expression of c-Myc ΔMBII mutant, which lacked the MBII domain and showed significantly reduced interaction with Skp2. As shown in supplementary Fig. S4, Gfi1 upregulated the expression of c-Myc ΔMBII mutant in Hela cells as efficiently as WT c-Myc, suggesting that Skp2 may not be a key player in Gfi1-mediated upregulation of c-Myc.

### Gfi1 stabilizes c-Myc protein independent of Miz-1

**Figure 4:**
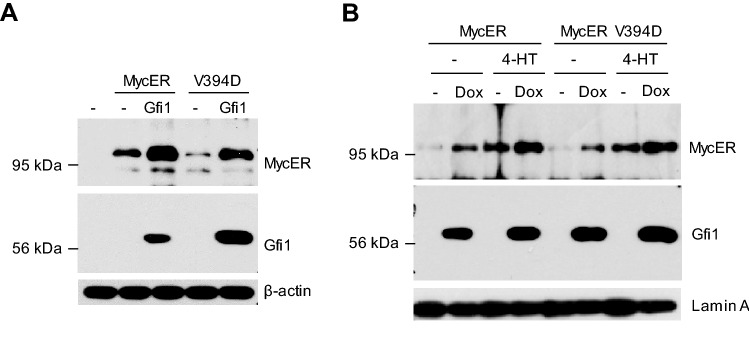
Gfi1 upregulates MycER protein independent of Miz-1. (**A**) Hela cells were transiently transfected with MycER, MycER V394D without or with Gfi1 prior to immunoblotting for the indicated proteins. (**B**) BaF3/MycER/Gfi1 and BaF/MycER-V394D/Gfi1 cells were treated with or without Dox and 4-HT for 24 h. Nuclear extracts were prepared and examined for the indicated proteins by Western blot analysis. Full-length blots are presented in Supplementary Fig. 9.

We previously showed that Gfi1 indirectly interacts with c-Myc through Miz-1^15,16^. Interestingly, Miz-1 has been shown to stabilize c-Myc^29^, raising the possibility that Gfi1 might stabilize c-Myc protein through Miz-1. We therefore examined the effect of Gfi1 on the expression of c-Myc-V394D mutant, which is defective in Miz-1 interaction, but retains the abilities to dimerize with Max and activate transcription^30,31^. As shown in Fig. 4, Gfi1 upregulated the level of c-Myc-V394D transiently expressed in Hela cells and the level of MycER V394D stably expressed in Ba/F3 cells. Thus, it appears that Gfi1-mediated upregulation of c-Myc was independent of Miz-1.

### Gfi1 upregulates the mRNA and protein levels of endogenous c-Myc

**Figure 5:**
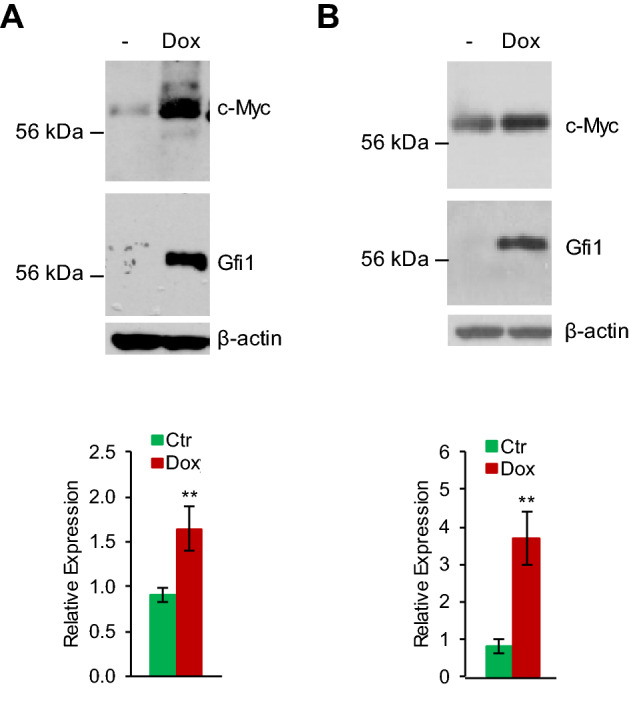
Gfi1 upregulates endogenous c-Myc at mRNA and protein levels. BaF/Gfi1 (**A**) and Ramos/Gfi1 (**B**) cells were untreated (Ctr) or treated with Dox for 24 h prior to evaluation of the expression of c-Myc protein by immunoblotting (upper panels) and mRNA by qRT-PCR (lower panels). Data are shown at mean ± SD. Full-length blots are presented in Supplementary Fig. 10.

Since Gfi1 increased the stability of endogenous c-Myc protein (Fig. 2A), we were interested to know whether Gfi1 regulated the expression of endogenous c-Myc. Indeed, Dox treatment of BaF/Gfi1 cells augmented the protein expression of endogenous c-Myc (Fig. 5A). Unexpectedly, however, Gfi1 also increased the mRNA level of endogenous c-Myc. To explore whether Gfi1 regulated c-Myc expression in lymphoma cells, we expressed the Dox-inducible Gfi1 in human Burkitt′s lymphoma Ramos cells (Ramos/Gfi1), which carried the characteristic t(8;14) chromosomal translocation that linked c-*MYC* to the immunoglobulin heavy-chain gene (IgH). As in BaF/Gfi1 cells, Gfi1 augmented the mRNA and protein levels of endogenous c-Myc in Ramos/Gfi1 cells (Fig. 5B).

**Figure 6:**
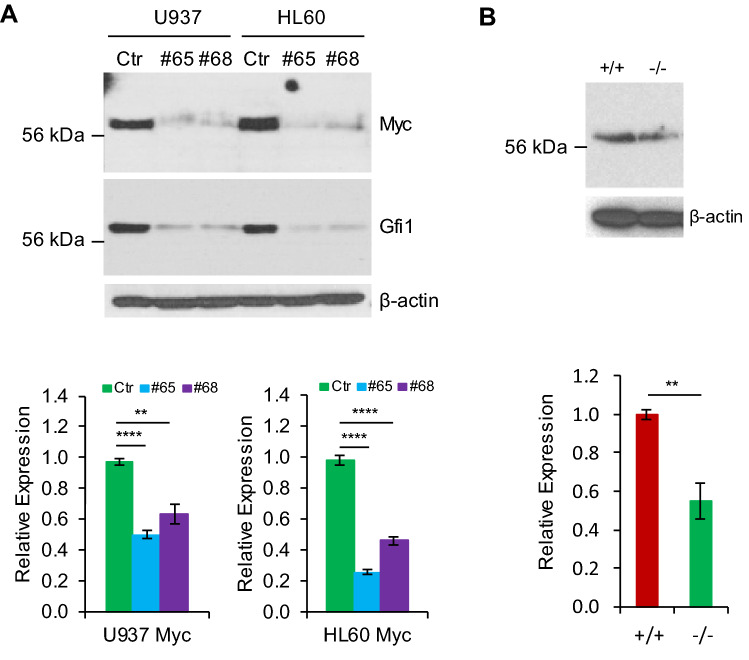
Gfi1 knockdown and deficiency downregulate c-Myc expression. (**A**) The expression of c-Myc protein (upper panels) and mRNA (lower panels) was examined in U937 and HL60 cells expressing the scrambled shRNA (Ctr) or shRNAs against Gfi1 (#65 and #68). (**B**) c-Myc protein and mRNA expression in Lin^-^ BM cells from Gfi1^+/+^ and Gfi1^-/-^ mice was examined. Data are shown as mean ± SD. Full-length blots are presented in Supplementary Fig. 11.

We previously showed that human leukemic HL60 and U937 cells expressed high levels of endogenous GFI1^15,16^. We examined the effect of GFI1 knockdown on c-MYC expression in these cells. As shown in Fig. 6A, GFI1 knockdown was associated with the decreased levels of c-MYC mRNA and protein. It appears that the effect of GFI1 knockdown on c-MYC protein expression was more dramatic than on mRNA expression in both cell lines. Furthermore, c-Myc mRNA and protein levels were decreased in Lin^-^ BM cells from *Gfi1*^*-/-*^ mice as compared to the levels in *Gfi1*^+*/*+^ BM cells (Fig. 6B), which was associated with decreased expression of c-Myc-activated target genes in *Gfi1*^*-/-*^ BM cells (supplementary Fig. S5). Together, these data indicate that Gfi1 upregulate c-Myc expression at both mRNA and protein levels.

### Gfi1 promotes c-Myc-driven cell proliferation

**Figure 7:**
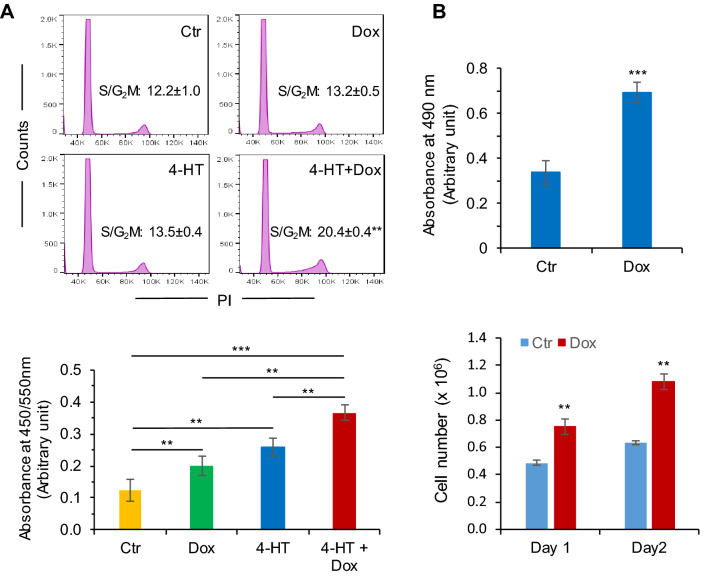
Gfi1 promotes c-Myc-driven cell proliferation. (**A**) BaF/MycER/Gfi1 cells were cultured in medium containing no growth factors in the absence or presence of Dox, 4-HT or both for 30 h. Cell cycle distributions were determined by flow cytometry based on DNA content (upper panels). ** denotes p < 0.01 as compared to cells untreated or treated with Dox or 4-HT alone. Cell proliferation was assessed using the BrdU incorporation assay (lower panel). (**B**) Ramos/Gfi1 cells were cultured in the absence (Ctr) or presence of Dox. Cell numbers were quantitated on day 3 by MTS assay (upper panel) or direct cell counting on the indicated days (lower panel). Data are shown as mean ± SD of three independent experiments.

c-Myc plays a central role in regulating cell proliferation and the oncogenic potential of Myc mainly derives from its ability to stimulate cell proliferation^32^. As Gfi1 increased c-Myc expression in Ba/F3 cells, we examined whether Gfi1 enhanced cell proliferation driven by c-Myc in BaF/MycER/Gfi1 cells, which were dependent on murine IL-3 for proliferation and survival. To test the effect of Gfi1 on Myc-driven cell proliferation, we cultured BaF/MycER/Gfi1 cells in medium containing no growth factors in the absence or presence of Dox, 4-HT or both. As shown in Fig. 7A, Dox or 4-HT alone had a modest but consistent effect on cell cycle progression. The combination of Dox and 4-HT significantly increased the proportion of cells in active phases of cell cycle G_2_/S/M. In BrdU cell proliferation assay, while Dox or 4-HT alone increased the proliferation of BaF/MycER/Gfi1 cells cultured in the absence of growth factors, Dox in combination with 4-HT led to a further increase in cell proliferation.

As shown in Fig. 5, Gfi1 upregulated the expression of c-MYC in Ramos Burkitt lymphoma cells in which c-MYC was overexpressed as a result of t(8;14) chromosomal translocation^33^. To assess the effect of Gfi1 on cell proliferation, Ramos/Gfi1 cells were cultured in the absence or presence of Dox. As shown in Fig. 7B, induction of Gfi1 expression with Dox led to a significant increase in cell proliferation, as determined by the MTS assay and direct cell counting. Together, these data indicate that Gfi1 acted in collaboration with c-Myc to drive cell proliferation.

## Discussion

Gfi1 has been shown to act in collaboration with c-Myc in lymphomagenesis, but the underlying molecular mechanisms remain incompletely understood. We previously showed that Gfi1 indirectly interacts with c-Myc through Miz-1 and collaborates with c-Myc in repressing *p15*^*INK4B*^, *p21*^*Cip1*^ and *p27*^*Kip1*^^15,16^. In this paper, we have shown that Gfi1 upregulates c-Myc expression at both mRNA and protein levels, and augments the effect of c-Myc on cell proliferation. We have further demonstrated that Gfi1 stabilizes c-Myc protein, likely by reducing its polyubiquitination. The ability of Gfi1 to upregulate c-Myc protein is dependent on its N-terminal SNAG and C-terminal ZF domains, but not on its DNA binding and repressor activities. Notably, although Miz-1 has been shown to stabilize c-Myc, Gfi1 was able to upregulate the expression of the c-Myc V394D mutant, which is defective in Miz-1 interaction, suggesting that Miz-1 may not be involved in Gfi1-mediated stabilization of c-Myc protein. Our data reveal a novel mechanism by which Gfi1 augments the biological function of c-Myc and may have implications for understanding the role of Gfi1 in Myc-mediated lymphomagenesis.

In addition to c-Myc, Gfi1 has been shown to stabilize another transcription factor GATA3, which was associated with reduced GATA3 polyubiquitination^34^. Interestingly, as in the case of c-Myc, the N-terminal SNAG domain of Gfi1, but not its repressor activity, is required for GATA3 stabilization. It has been shown that the SNAG domain of Gfi1 is required for its nuclear localization^23^ and to interact with the histone demethylase LSD1 and the corepressor Co-REST^35^. Notably, substitution of the proline at position 2 for alanine (P2A) disrupts Gfi1 interactions with LSD1 and Co-REST without altering its nuclear localization^23^. Thus, it appears that Gfi1 needs to be in the nucleus to stabilize c-Myc and GATA3, which are also nuclear proteins. It was not examined in the previous study whether the C-terminal ZF domains are required for GATA3 stabilization.

It is unknown how Gfi1 influences c-Myc polyubiquitination. The fact that the C-terminal ZF domains of Gfi1, which are required for its polyubiquitination and degradation^24^, are also required for c-Myc upregulation raises the possibility that Gfi1 and c-Myc might compete for binding to a common E3 ubiquitin ligase(s). Interestingly, both Gfi1 and c-Myc have been identified as the substrates of Fbxw7^25^. However, our data indicate that Fbxw7 may not play a key role in Gfi1-mediated regulation of c-Myc protein. Skp2 is another major regulator of c-Myc protein stability. We have shown that Gfi1 efficiently upregulated the expression of c-Myc ΔMBII mutant, which is significantly impaired in its ability to interact with Skp2, making it less likely that Skp2 is the major player in c-Myc upregulation by Gfi1. It remains to be determined whether other E3 ubiquitin ligases or other mechanisms are involved in Gfi1-mediated c-Myc upregulation.

The mechanism by which Gfi1 upregulates the mRNA level of c-Myc is also unknown. Gfi1 has been shown to function mainly as a transcriptional repressor. However, the possibility cannot be excluded that Gfi1 may bind to *c-Myc* promoter to activate its transcription. Interestingly, analysis of the ChIP-seq data (GSE31657) submitted by Möröy’s research group^12^ indicates that Gfi1 may bind to the promoters of *c-Myc* in murine hematopoietic progenitor cells (data not shown). An alternative possibility is that Gfi1 may repress a negative regulator of *c-Myc*, such as a transcription factor or microRNA, leading to upregulation of c-Myc mRNA. In this aspect, it is of note that Gfi1 has been shown to repress microRNA-196b (*miR-196b*) and *miR-*21^36^, and miR-196b has been shown to suppress c-Myc mRNA expression^37^. Further studies are needed to address how Gfi1 upregulates c-Myc mRNA expression.

## Materials and methods

### Cell lines and cell culture

Murine Pro-B Ba/F3 and myeloid 32D cells expressing the doxycycline (Dox)-inducible Gfi1 lentiviral construct pPMPrtTA-Gfi1-GFP, and human myeloid leukemic cell lines U937 and HL-60 expressing the shRNAs against GFI1 as well as Hela cells have been described previously^15,16,38^. Ba/F3 cells were maintained in RPMI-1640 medium supplemented with 4% fetal bovine serum (FBS), 4% WEHI-3B cell-conditioned media (WEHI-CM) as a crude source of murine interleukin-3 and 1% penicillin/streptomycin (P/S). 32D cells were maintained in RPMI-1640 with 10% FBS, 10% WEHI-CM and 1% P/S. HL-60, U937 and Human Burkitt lymphoma Ramos cells were cultured in RPMI-1640 medium supplemented with 10% FBS and 1% P/S. The parental and the *FBXW7*^*-/-*^ HCT116 cells were kindly provided by Dr. Shi-Yong Sun (Emory University School of Medicine) with permission from Dr. Bert Vogelstein (Johns Hopkins Medical School). HCT and HeLa cells were maintained in Dulbecco’s modified Eagle’s medium containing 10% FBS and 1% P/S. All cells were grown in humidified incubator at 37 ºC with 5% CO2.

### Reagents and expression constructs

Antibodies against Gfi1 (N-20), c-Myc (N262), C/EBPε (C-22), Miz-1 (N17) and His tag (H-15) were purchased from Santa Cruz Biotechnolgy (Santa Cruz, CA). Antibodies against Flag tag and β-actin were from Sigma (Saint Louis, MO). Anti-HA antibody and anti-lamin A antibody were obtained from Cell Signaling (Beverly, MA) and Abcam (Cambridge, MA), respectively. *Trans*IT-LT1 Transfection Reagent was purchased from Mirus (Madison, WI). BrdU cell proliferation assay kit was purchased from Millipore (Burlington, MA).

The expression constructs for c-Myc, Flag-tagged Gfi-1 and the different Gfi1 mutants used in transient transfection have been described before^15,16^. c-Myc T58A mutant was generated by site-directed mutagenesis and confirmed by DNA sequencing.

### Stable transfection

Ba/F3 and 32D cells were transfected with the retroviral vector pBabe-puro containing the cDNA encoding c-Myc/estrogen receptor ligand binding domain fusion protein (MycER) or MycER V394D (kindly provided by Dr. Martin Eilers) by electroporation and selected in puromycin (1.5 µg/ml). Individual clones expressing the MycER proteins were obtained by limiting dilution, pooled and subsequently transduced with pPMPrtTA-Gfi1-GFP, essentially as described^38^.

### Preparation of whole cell and nuclear extracts, and Western blot analysis

Cells were lysed in SDS lysis buffer (1% SDS, 50 mM Tris–HCl [pH 8.0] and 10 mM EDTA [pH 8.0]). Nuclear extracts were prepared as described^15^. Proteins were separated by SDS-PAGE prior to transfer onto polyvinylidenedifluoride (PVDF) membranes. The membranes were incubated with the appropriate antibodies and signals were detected by enhanced chemiluminescence.

### Mice, bone marrow cell isolation

Gfi1 knockout mice^39^ were bred and housed in the animal facility at The University of Toledo. All experiments using mouse BM cells were approved by the Institutional Animal Care and Use Committee (IACUC) of The University of Toledo. All experiments were performed in accordance with relevant guidelines and regulations. Bone marrow cells were isolated from 6- to 8-week-old C57BL/6 WT and Gfi1 mutant mice, essentially as previously described^40^. Lin^-^ cells were purified using the mouse Lineage Cell Depletion kit (Miltenyi Biotec).

### Real-time reverse transcription polymerase chain reaction (qRT-PCR)

Total RNA was extracted using TRIzol reagent (Invitrogen) and cDNA was synthesized using the GoScript Reverse Transcription System and Oligo(dT)15 primer (Promega, Madison, WI). qRT-PCR was performed using the SsoFast EvaGreen Supermix kit (Bio-Rad) and the relative levels of mRNAs for the different myeloid differentiation markers were normalized to GAPDH mRNA expression. The following primer sets were used: *MycER*, forward 5′-TGTCCATTCAAGCAGACGAG-3′ and reverse 5′-AAGGACAAGGCAGGGCTATT-3′; mouse *c-Myc*, forward 5′-CCTAGTGCTGCATGAGGAGA-3′ and reverse 5′-TCCACAGACACCACATCAATTT-3′; human *c-MYC*, forward 5′-TCAAGAGGCGAACACACAAC-3′ and reverse 5′-GGCCTTTTCATTGTTTTCCA-3′; mouse *Odc,* forward 5′-CTGATCCTGAGACCTTCGTTC-3′ and reverse 5′-AGAGCTGGGTTGATTACACTG-3′; mouse *Rcl*, forward 5′-TGGGTGTTGGCTACGAATTG-3′ and reverse 5′-AAAGTACCGATGGAGCATGG-3′; mouse *E2f2*, forward 5′-ACCTGACCGAAGATAATGCC-3′ and reverse 5′-ACTGTCTGCTCCTTGAAGTTG-3′. The experiments were conducted in triplicate and all experiments were repeated at least once.

### Cell cycle analysis

BaF/MycER/Gfi1 cells were cultured in medium containing no growth factors without or with 4-HT, Dox or both for 30 h. The cells were fixed in 70% ethanol at -20 °C for 24 h, washed and then treated with RNase A (0.5 µg/ml) for 30 min at 37 °C prior to labeling with propidium iodide (50 µg/ml). Cell cycle distributions were examined by flow cytometry on an LSR Fortessa Cell Analyzer (BD Biosciences) and data were analyzed with FlowJo cell cycle Watson (Pragmatic) model. Results are based on three independent experiments.

### BrdU cell proliferation assay

BaF/MycER/Gfi1 cells were seeded in triplicate in 96-well plates in medium containing no growth factors in the absence or presence of 4-HT, Dox or both for 24 h, followed by incubation with BrdU for 6 h. BrdU incorporation was determined according to the manufacturer’s instructions. The plates was read using the SpectraMax iD5 Multi-Mode Microplate Reader (Molecular Devices) at a dual wavelength of 450/550 nm.

### MTS assay

The experiments were performed according to the manufacturer’s instructions (CellTiter 96 AQueous Non-Radioactive Cell Proliferation Assay; Promega, Madison, WI), as described before^38^. Ramos cells (2 × 10^4^) were incubated in triplicate in 100 µl of RPMI 1640 medium in 96-well plates in the absence or presence of Dox (1 µg/ml) for 3 days. CellTiter 96 AQ_ueous_ One Solution Reagent was added to each well and the plates were read 4 h later at 490-nm wavelength using Lmaxluminometer (Molecular Devices, Sunnyvale, CA).

### Ubiquitination assay

Hela cells were transfected with His-tagged ubiquitin, HA-tagged c-Myc without or with Gfi1 using Mirus Transfection Reagent. After incubation for 30 h, cells were treated with MG132 (40 µM) for 6 h and lysed in SDS lysis buffer. Samples were boiled for 10 min, sonicated briefly and diluted 1:10 with dilution buffer. After centrifugation, samples were subjected to immunoprecipitation using the anti-HA antibody under denaturing conditions prior to Western blot analysis.

### Statistics

All statistical analyses were performed using GraphPad Prism software (GraphPad Software, La Jolla, CA, USA). Data are presented as mean ± SD with * indicating p < 0.05, ** < 0.01, and *** < 0.001 in all figures.

## Supplementary information

Supplementary information.

## Data Availability

No datasets were generated or analyzed during the current study.
