# Supplementary material for: Gfi1 upregulates c-Myc expression and promotes c-Myc-driven cell proliferation
Source: Sci Rep. 2020 Oct 13;10:17115. doi: 10.1038/s41598-020-74278-4 (PMC7554040; doi:10.1038/s41598-020-74278-4)
Supplement: Supplementary file 1 — Supplementary information. [file 41598_2020_74278_MOESM1_ESM.pdf]

Gfi1 Upregulates c-Myc Expression and Promotes c-Myc-Driven Cell Proliferation

Yangyang Zhang and Fan Dong[1]

Department of Biological Sciences, University of Toledo, Toledo, Ohio 43606

[1] Correspondence and requests for materials should be addressed to F.D. (email: **fan.dong@utoledo.edu**)

Supplementary Figure Legends

Supplementary Figure S1. The expression of AML1, C/EBPε, STAT5 and Miz-1 is not affected by Gfi1. Hela cells were transfected with the expression constructs for Flag-tagged AML1, C/EBPε, Flag-tagged STAT5 and Miz-1 alone or together with Gfi1. The expression of the indicated proteins was examined by Western blot analysis. Note that AML1 was expressed as proteins of variable sizes as have been seen in hematopoietic cells. Full-length blots are presented in Supplementary Figure 12.

Supplementary Figure S2. Comparison of doxycycline (Dox)-induced expression of Gfi1 in BaF/Gfi1 and expression of endogenous Gfi1 in HL-60 and U937 cells. BaF/Gfi1 cells were either untreated or treated with (Dox) for 24 hours before the cells were lysed for Western blot analysis. Full-length blots are presented in Supplementary Figure 13.

Supplementary Figure S3. Fbxw7 is not required for c-Myc upregulation by Gfi1. (A) Hela cells were transfected with c-Myc T58A without or with Gfi1. (B) WT and *FBXW7*$^{-/-}$ HCT 116 cells were transfected with increasing amounts of c-Myc without or with Gfi1. Whole cell extracts were prepared and the expression of indicated proteins was examined by Western blot analysis. Full-length blots are presented in Supplementary Figure 14.

Supplementary Figure S4. Deletion of c-Myc MBII has no effect on c-Myc upregulation by Gfi1. Hela cells were transfected with WT c-Myc or c-Myc ΔMBII without or with Gfi1 prior to immunoblotting for the indicated proteins. Full-length blots are presented in Supplementary Figure 15.

Supplementary Figure S5. Gfi1 deficiency downregulates the expression of c-Myc target genes. The mRNA levels of c-Myc target genes as indicated in Lin^- BM cells from $Gfi1^{+/+}$ and $Gfi1^{-/-}$ mice were examined by qRT-PCR. Data are shown at mean ± SD.

Supplementary Figure S6. Original full-length blots of Figure 1.

Supplementary Figure S7. Original full-length blots of Figure 2.

Supplementary Figure S8. Original full-length blots of Figure 3.

Supplementary Figure S9. Original full-length blots of Figure 4.

Supplementary Figure S10. Original full-length blots of Figure 5.

Supplementary Figure S11. Original full-length blots of Figure 6.

Supplementary Figure S12. Original full-length blots of Supplementary Figure S1.

Supplementary Figure S13. Original full-length blots of Supplementary Figure S2.

Supplementary Figure S14. Original full-length blots of Supplementary Figure S3.

Supplementary Figure S15. Original full-length blots of Supplementary Figure S4.

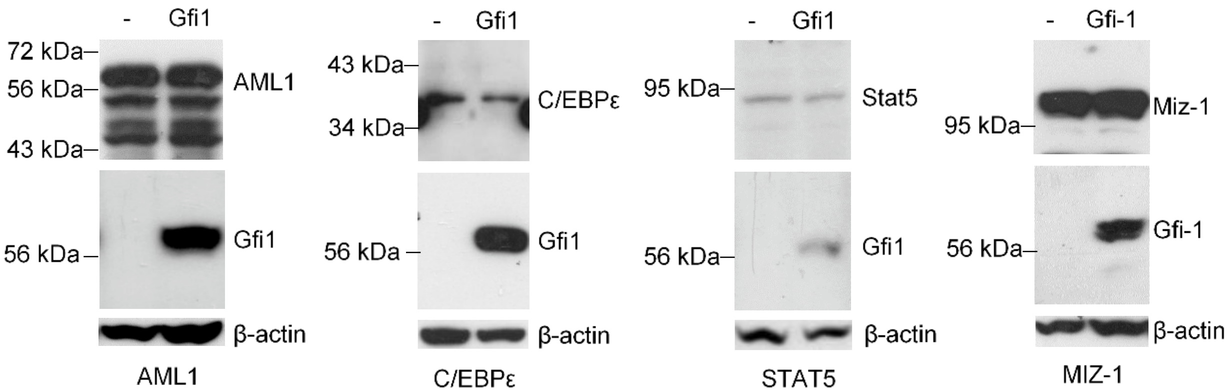

Supplementary Figure S1

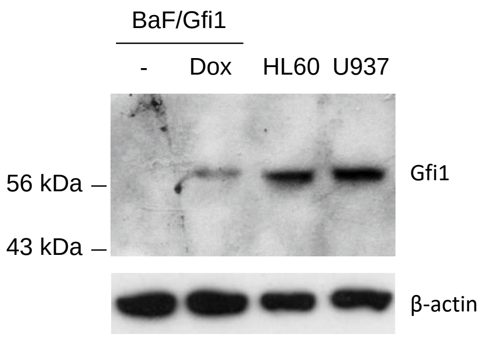

Supplementary Figure S2

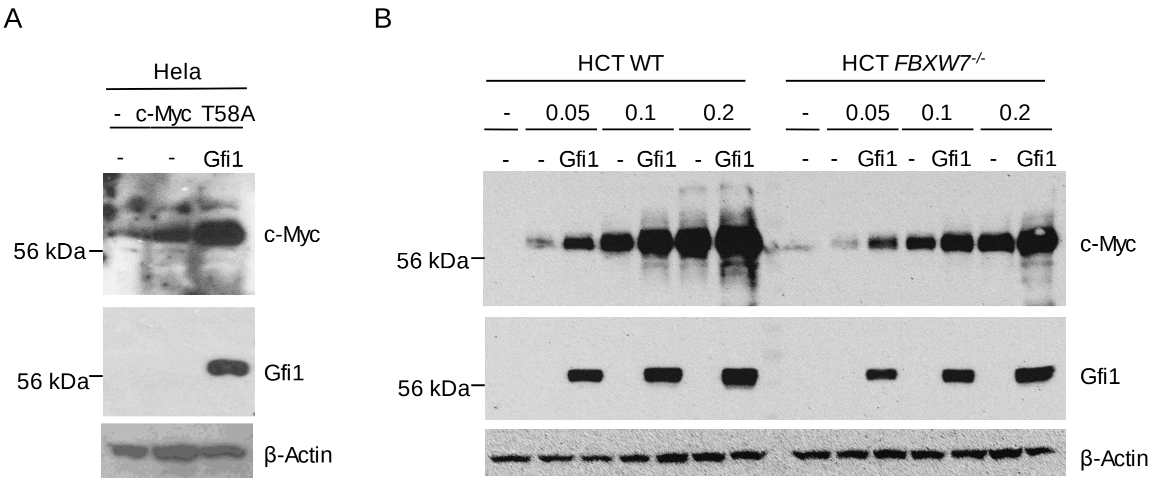

Supplementary Figure S3

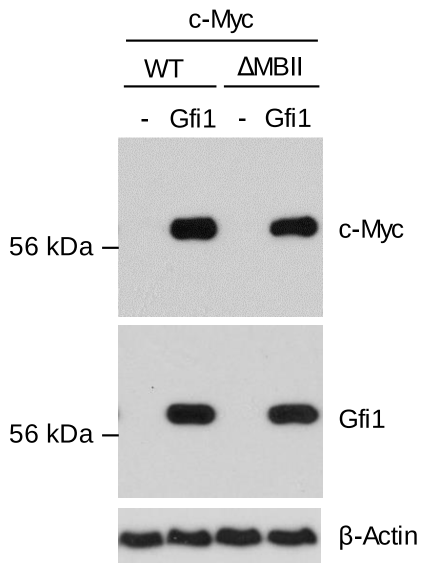

Supplementary Figure S4

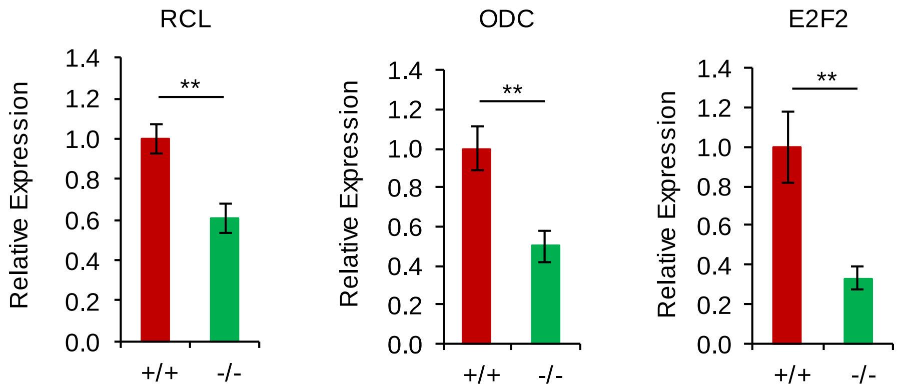

Supplementary Figure S5

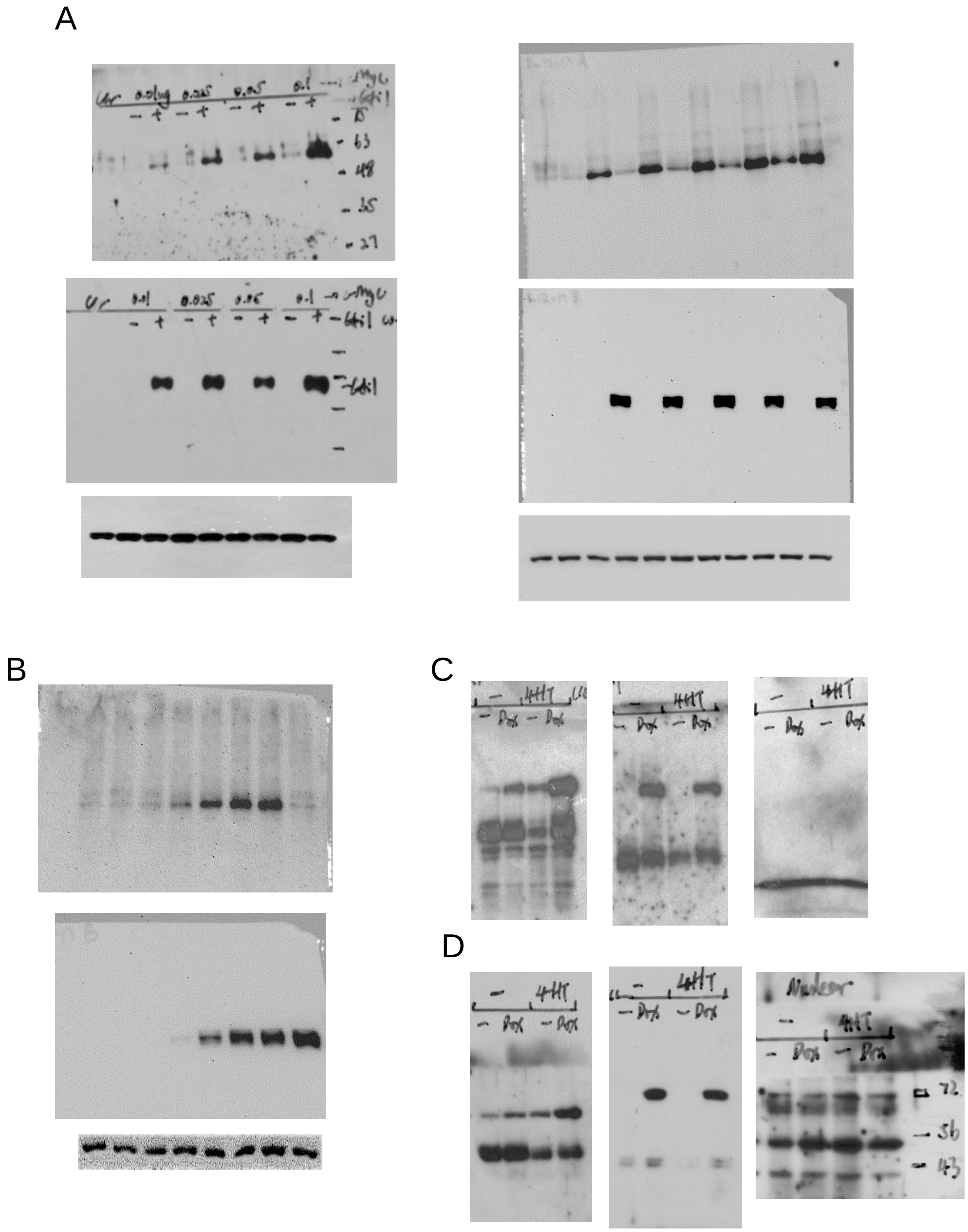

Supplementary Figure S6

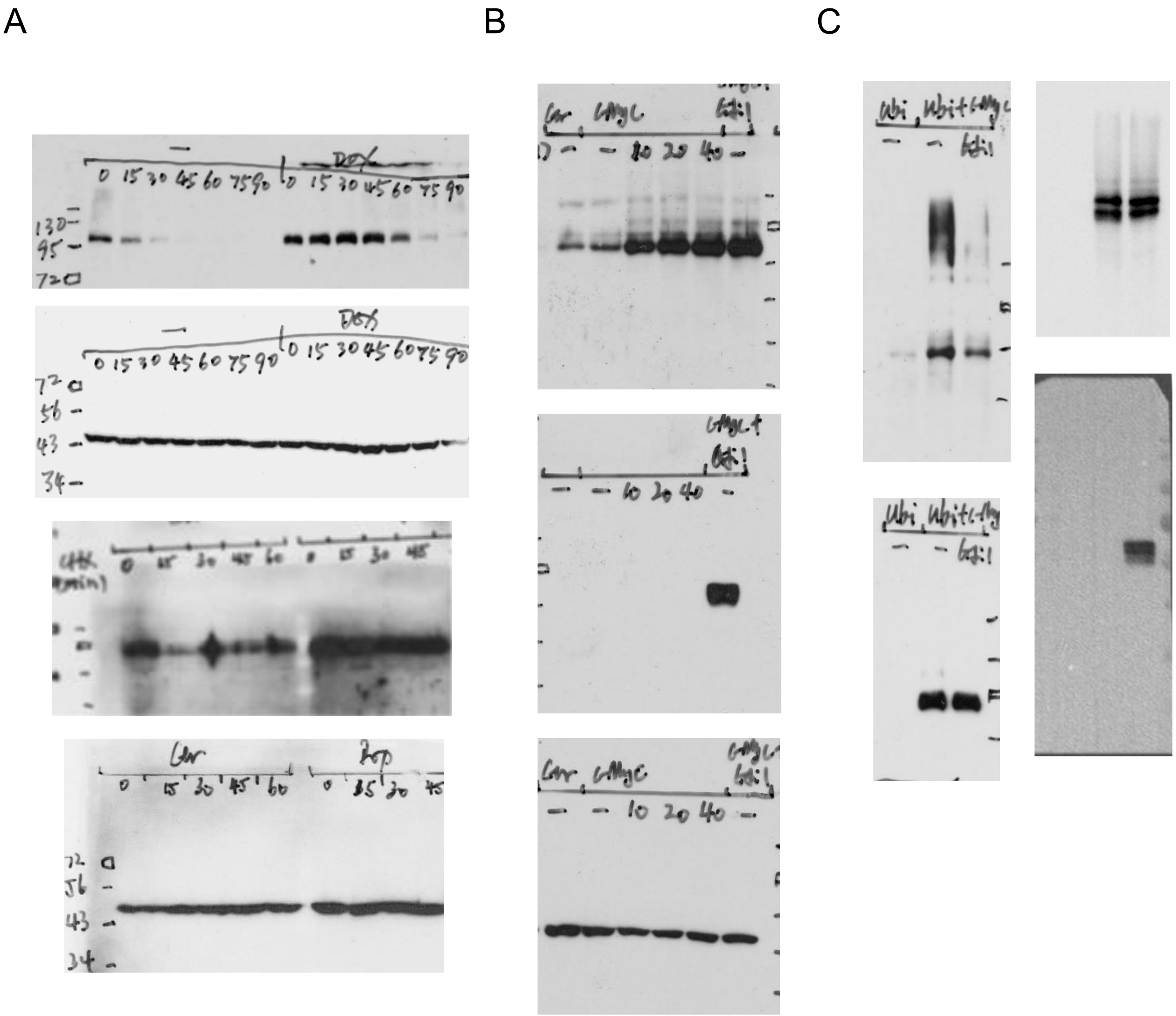

Supplementary Figure S7

A

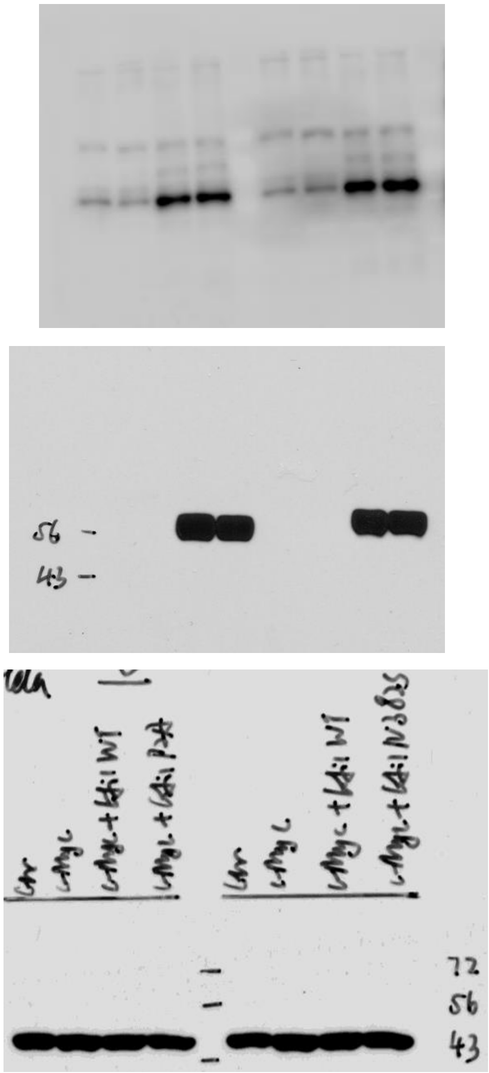

C

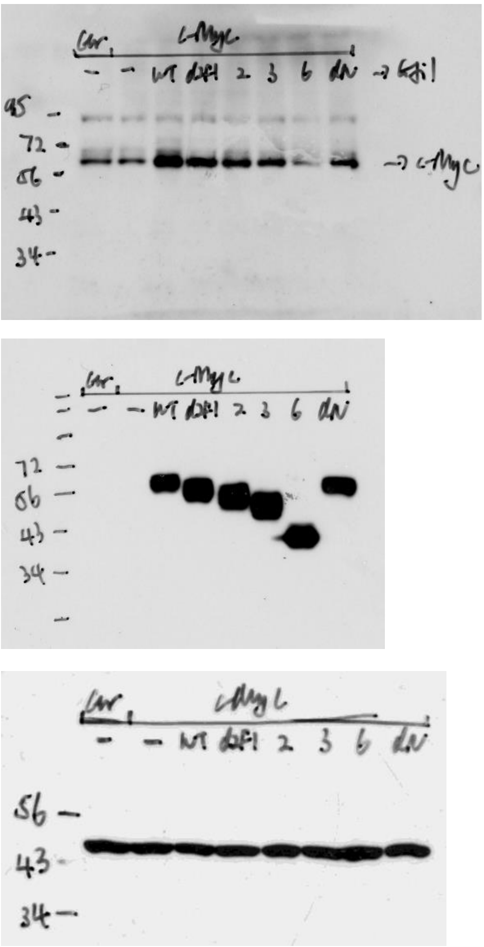

Supplementary Figure S8

A B

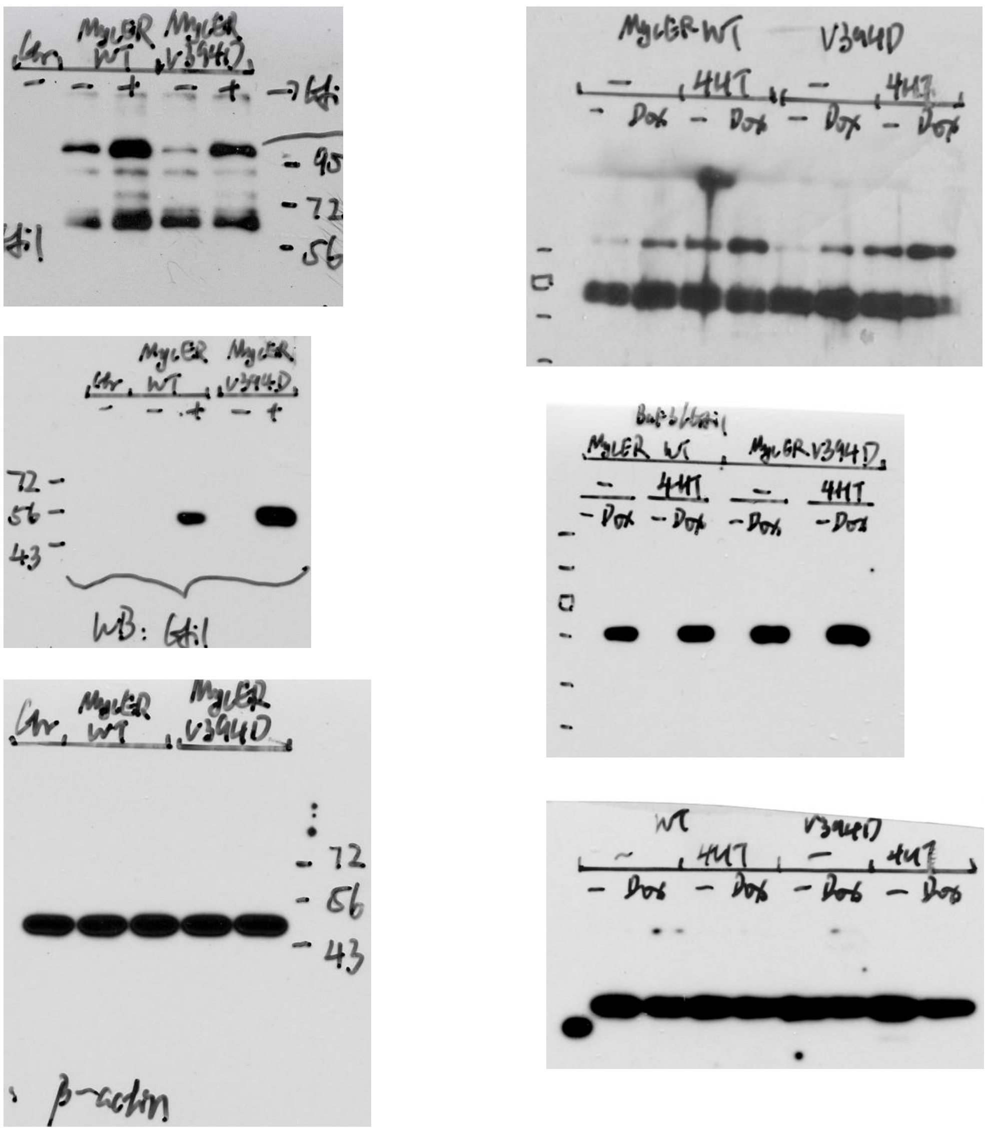

Supplementary Figure S9

A B

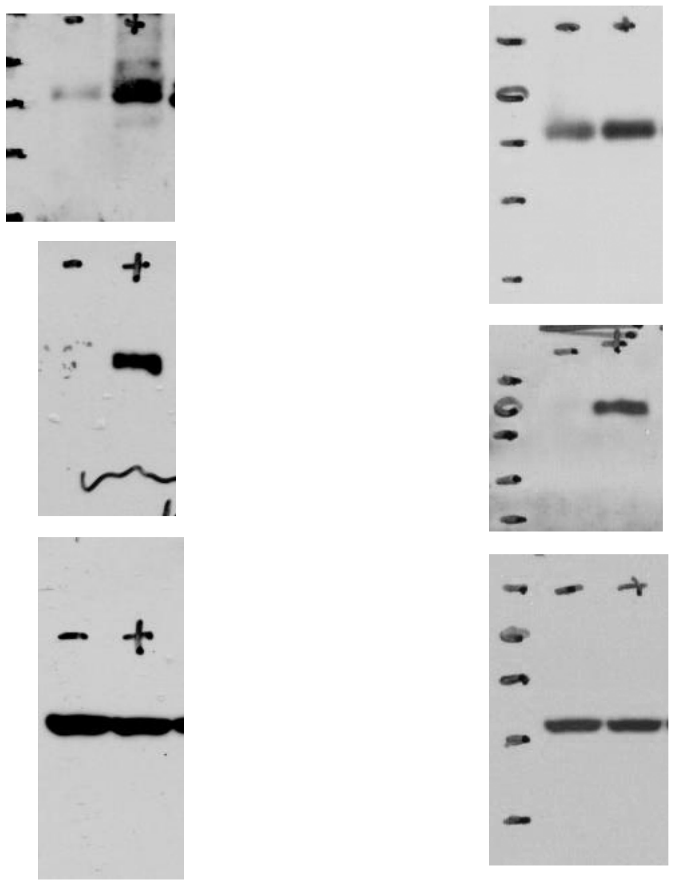

Supplementary Figure S10

A B

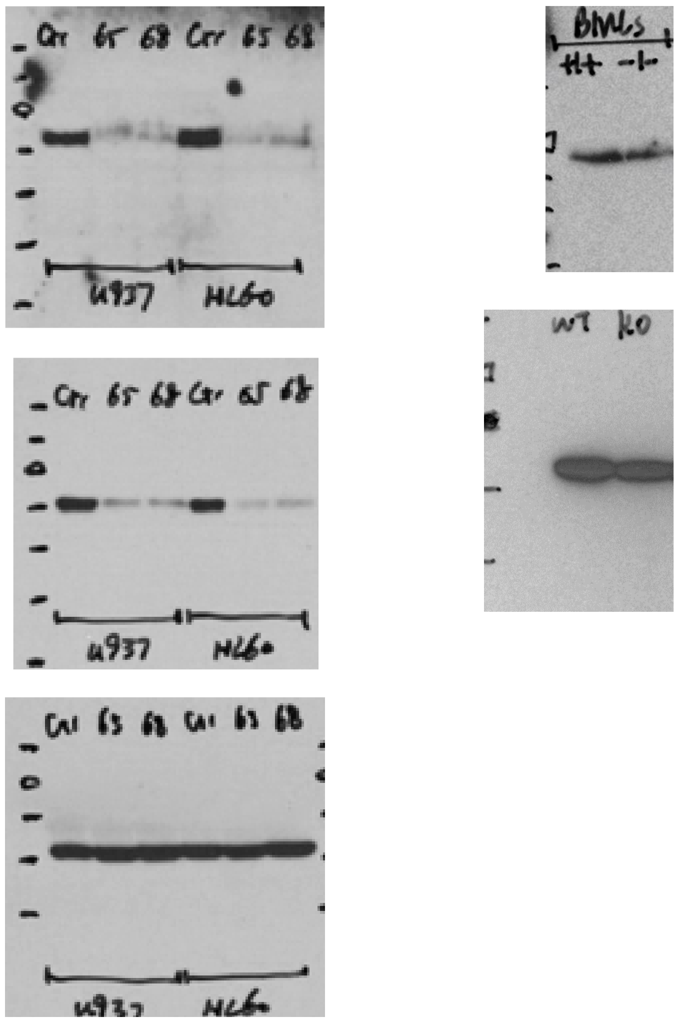

Supplementary Figure S11

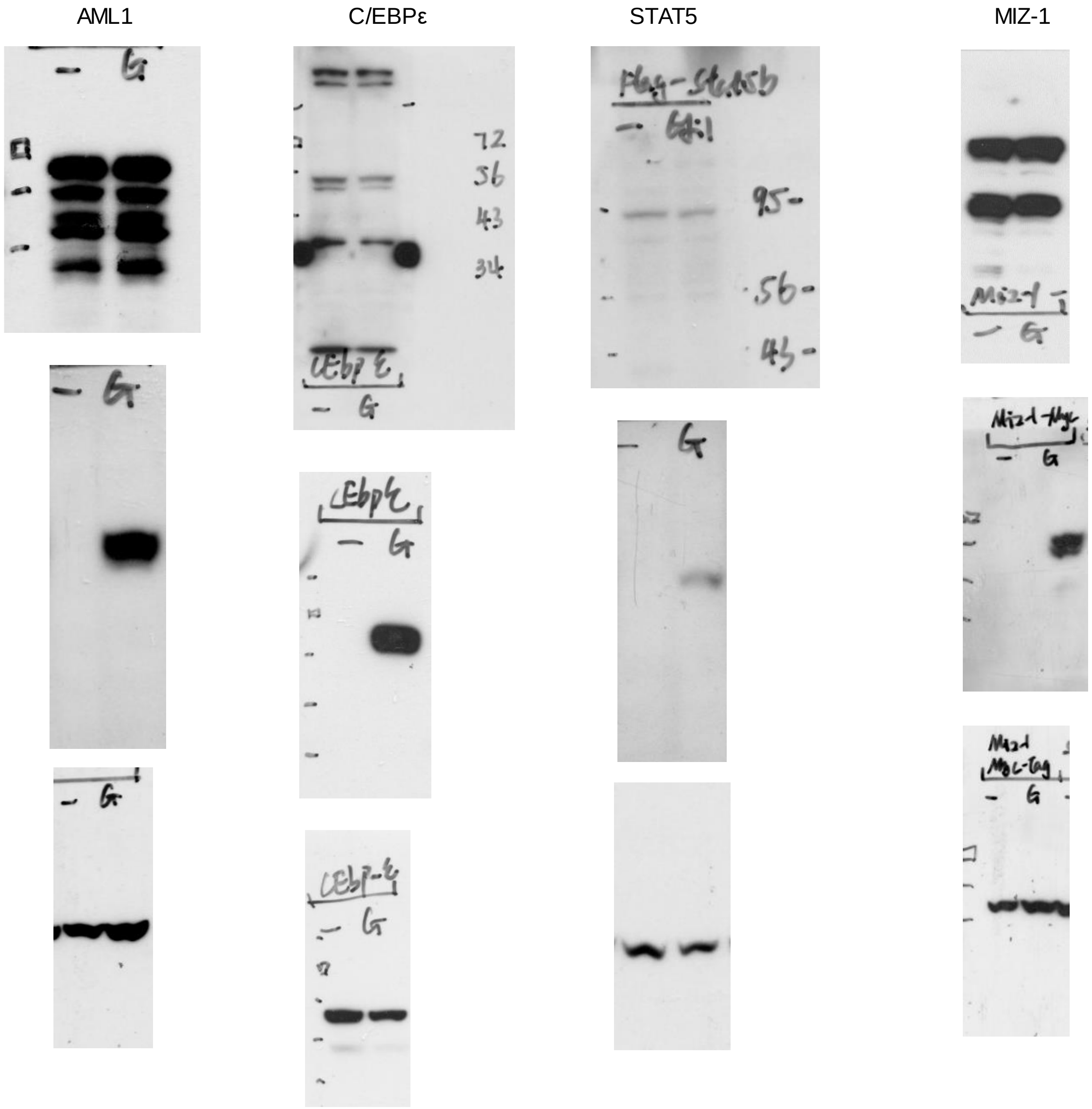

Supplementary Figure S12

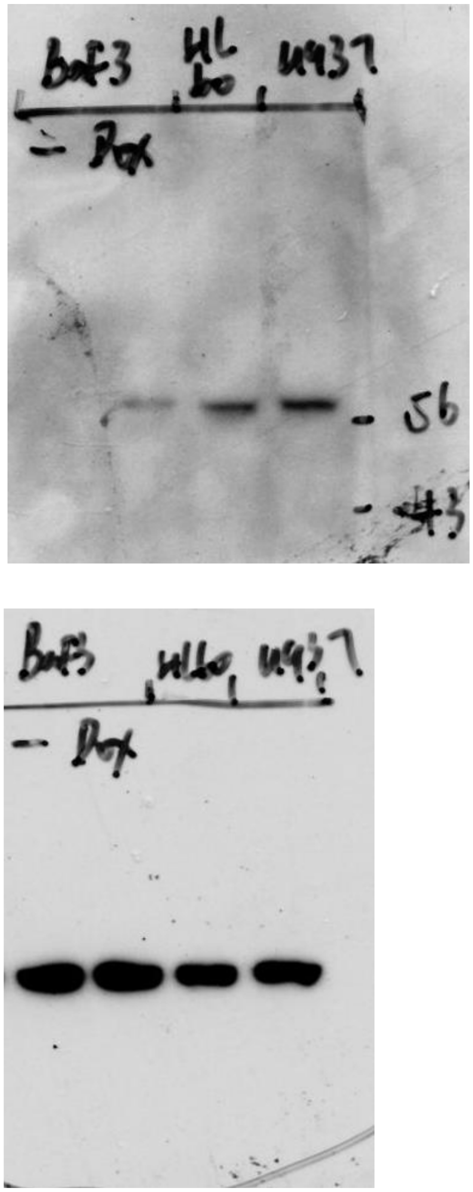

Supplementary Figure S13

A

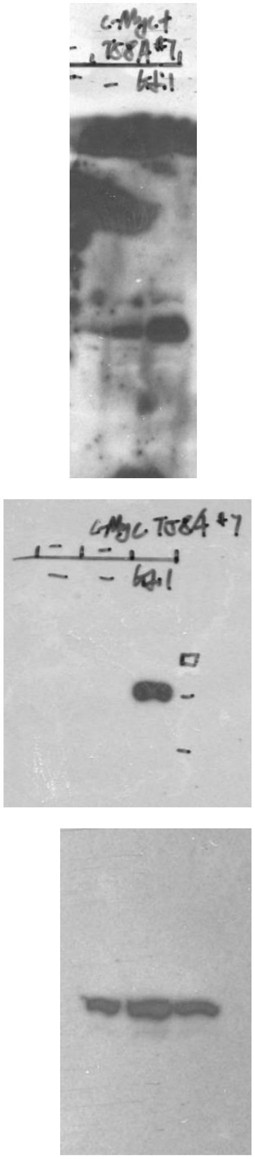

B

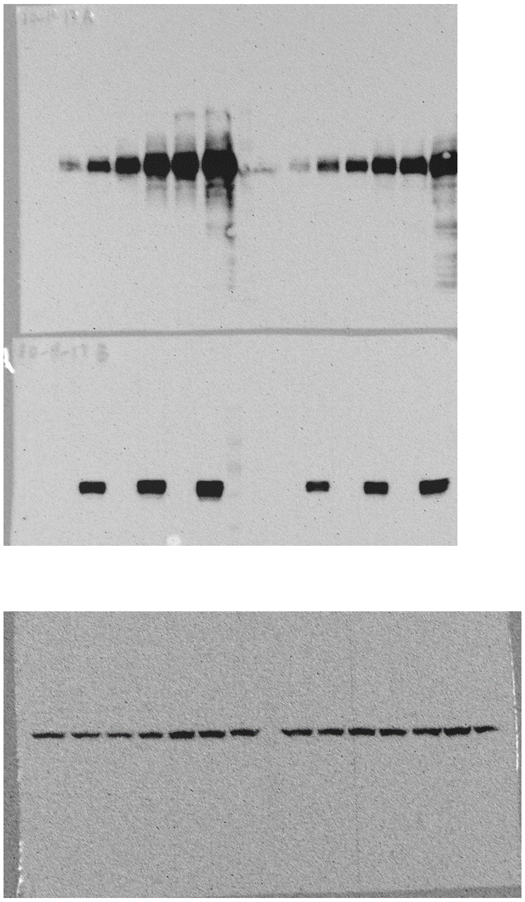

Supplementary Figure S14

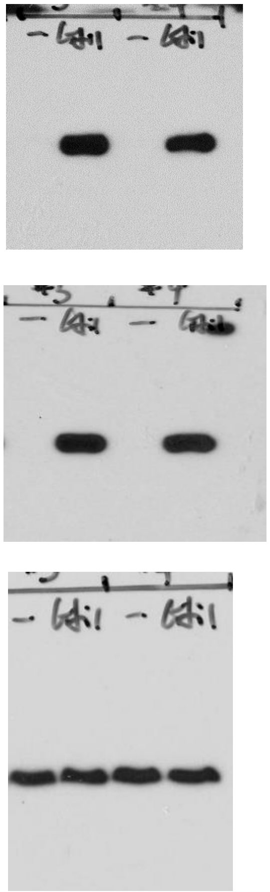

Supplementary Figure S15
